# Bladder cancer, a review of the environmental risk factors

**DOI:** 10.1186/1476-069X-11-S1-S11

**Published:** 2012-06-28

**Authors:** Silvia Letašiová, Alžbeta Medveďová, Andrea Šovčíková, Mária Dušinská, Katarína Volkovová, Claudia Mosoiu, Alena Bartonová

**Affiliations:** 1 Institute of Biochemistry, Nutrition and Health Protection, Faculty of Chemical and Food Technology, Slovak University of Technology, Radlinského 9, SK-812 37 Bratislava, Slovak Republic; 2 Research Base of Slovak Medical University, Limbová 12, SK-833 03, Bratislava, Slovak Republic; 3 Institute of Food Bioresources, 6 Dinu Vintila Street, 021102 Bucharest 2, Romania; 4 NILU-Norwegian Institute for Air Research, POB 100, N-2027 Kjeller, Norway

## Abstract

**Background:**

Many epidemiological studies and reviews have been performed to identify the causes of bladder cancer. The aim of this review is to investigate the links between various environmental risk factors and cancer of the bladder.

**Methods:**

A systematic literature search was performed using PubMed, Science Direct, Scopus, Scholar Google and Russian Google databases to identify reviews and epidemiological studies on bladder cancer risk factors associated with the environment published between 1998 and 2010. Only literature discussing human studies was considered.

**Results:**

Smoking, mainly cigarette smoking, is a well known risk factor for various diseases, including bladder cancer. Another factor strongly associated with bladder cancer is exposure to arsenic in drinking water at concentrations higher than 300 µg/l. The most notable risk factor for development of bladder cancer is occupational exposure to aromatic amines (2-naphthylamine, 4-aminobiphenyl and benzidine) and 4,4'-methylenebis(2-chloroaniline), which can be found in the products of the chemical, dye and rubber industries as well as in hair dyes, paints, fungicides, cigarette smoke, plastics, metals and motor vehicle exhaust. There are also data suggesting an effect from of other types of smoking besides cigarettes (cigar, pipe, Egyptian waterpipe, smokeless tobacco and environmental tobacco smoking), and other sources of arsenic exposure such as air, food, occupational hazards, and tobacco. Other studies show that hairdressers and barbers with occupational exposure to hair dyes experience enhanced risk of bladder cancer. For example, a study related to personal use of hair dyes demonstrates an elevated bladder cancer risk for people who used permanent hair dyes at least once a month, for one year or longer.

**Conclusion:**

Smoking, in particular from cigarettes, exposure to arsenic in drinking water, and occupational exposure to aromatic amines and 4,4'-methylenebis(2-chloroaniline) are well known risk factors for various diseases including bladder cancer. Although the number of chemicals related to occupational exposure is still growing, it is worth noting that it may take several years or decades between exposure and the subsequent cancer.

## Background

Bladder cancer is the 10th most common cancer worldwide, with the highest rates reported in Europe, North America and Australia, and accounting for an estimated 261 000 new cases diagnosed and 115 000 deaths each year; by comparison, relatively low rates are found in the Far Eastern countries [1,2]. In Europe, bladder cancer is the 5th most commonly diagnosed cancer type and the 9th leading cause of cancer mortality [2]. It affects men more frequently than women [3]. Typical of solid tumours, bladder cancer incidence increases with age. Tumours of the bladder rarely occur before the age of 40 – 50, arising most commonly in the seventh decade of life [4,5]. The median ages at diagnosis are 69 years for men and 71 for women [6].

Histologically, most cases of bladder cancer are transitional cell carcinomas (90 %); 70 % of these are superficial and papillary subtypes [7]. The less common types are squamous cell carcinoma (3 - 5 %); adenocarcinoma (0.5 to 2 %); small cell carcinoma (less than 0.5 %); and sarcoma, carcinosarcoma/sarcomatoid tumours, paraganglioma, melanoma and lymphoma (less than 0.1 %) [8]. Haematuria, i.e., frequent urination and pain during urination, are the most common symptoms of bladder cancer [4].

The aim of this review is to investigate the links between environmental risk factors and bladder cancer. Examples include the effect of different types of smoking (cigarette, cigar, pipe and environmental tobacco smoking); the effect of arsenic in drinking water, air, food, occupational hazards, and tobacco; and the effect of occupational exposure to aromatic amines (2-naphthylamine, 4-aminobiphenyl and benzidine) and 4,4'-methylenebis(2-chloroaniline) that can be found in chemical, dye and rubber industries as well as in hair dyes, paints, fungicides, cigarette smoke, plastics, metal and motor vehicle exhaust in relation to bladder cancer.

## Methods

A systematic literature search was performed using PubMed, Science Direct, Scopus, Scholar Google and Russian Google databases to identify reviews and epidemiological studies on bladder cancer risk factors associated with the environment published between 1998 and 2010. Only literature discussing human studies was considered.

## Results

### Smoking

Smoking is a well known risk factor of chronic lung disease, heart disease and various types of cancer including bladder cancer [9,10]. Several epidemiological studies and reviews describe the impact of cigarette, cigar, pipe and environmental tobacco smoking.

Cigarette smoking is the primary risk factor for bladder cancer [3,6,11]. In a recent meta-analysis of 43 published case-control and cohort studies, Zeegers et al. [12] concluded that current cigarette smokers have an approximately threefold higher risk of bladder cancer than non-smokers. In a combined analysis of 11 case-control studies from six European countries, risk for bladder cancer increased with duration of smoking (number of years smoked) and intensity of smoking (number of cigarettes smoked per day) [3,13,14]. The age- and gender-adjusted summary odds ratios for current and former cigarette smokers compared with those for non-smokers were 3.33 (95 % confidence interval (CI), 2.63 – 4.21) and 1.98 (95 % CI 1.72 – 2.29), respectively [4]. The Netherlands Cohort Study the associations between cigarette smoking and bladder cancer risks were studied in detail [15]. Zeegers et al. [15] found out that the tar and nicotine content of cigarettes, and filter-tip usage were only weakly associated with bladder cancer risk. Cancer of the urinary bladder has a relative risk associated with tobacco use of 3.0; the relative risk for pancreas cancer associated with tobacco use is 2.0 – 4.0 [11].

Given convincing evidence for a substantially increased risk of bladder cancer for cigarette smokers, Zeegers et al. [4] calculated the etiologic fraction of cigarette smoking which accounts for 23 % of all female bladder cancer, whereas in men 50 % of the cancer is attributed to cigarette smoking.

Risk depends on the method of tobacco smoking: pure cigarette smokers were at higher risk (95 % CI 2.9 – 4.2) than pure pipe smokers (95 % CI 1.2 – 3.1) or pure cigar smokers (95 % CI 1.6 – 3.5) [16]. The effect of cigar smoking may be limited to people who inhale [17]. Furthermore, Iscovich et al. [18] suggested that users of black tobacco have a two to three times higher risk than users of Virginia tobacco. In the Netherlands Cohort Study, Zeegers et al. [4] confirmed a substantially increased risk of bladder cancer for current cigar (95 % 1.68 – 4.93) and pipe smokers (95 % 1.67 – 5.50) versus those who have never smoked tobacco. However, the evidence of an association between bladder cancer risk and Egyptian waterpipe and smokeless tobacco use is rarely reported [3].

The precise mechanism by which cigarette, cigar and pipe smoking causes bladder cancer has yet to be determined. Zeegers et al. [4] assumed that the risk of bladder cancer is related to some of the large number of chemicals present in smoke, such as 2-naphthylamine and 4-aminobiphenyl, leading candidates for the specific etiologic agents. On the other hand, cigar and pipe smokers inhale less than cigarette smokers, which can explain an increased cancer incidence for cigar and pipe smokers in local areas of the human body (such as head cancer, neck cancer) and no increased risk through a systemic effect (such as bladder cancer) [4].

The relationship of environmental tobacco smoking has been investigated only in a few studies [4,19,20]. Kabat et al. [21] reported data from a population-based US case control study where no significant risk of bladder cancer in either sex was seen when comparing 84 non-smoking cases and 266 hospital controls [19]. In a large Japanese prospective study no significant increased risk was observed in the wife associated with the husband's smoking [22]. A population-based case-control study was conducted in Canada [23]. Risk of bladder cancer was not increased in relation to environmental tobacco smoking exposure at home or at work [19].

There is good evidence that stopping smoking reduces the rate of recurrence for many cancers [24]. For bladder cancer, Aveyard et al. [25] examined 15 studies that showed evidence that continued smoking or a lifetime of smoking constitutes a moderate risk factor for recurrence and death, and that stopping smoking could favourably change this. However, the evidence base for this is weak because of the methodological shortcomings and because most results of the studies were not statistically significant [25].

### Arsenic in drinking water

Arsenic, a naturally occurring metalloid found in air, soil and water, exists in both organic and inorganic states. Whereas organic forms are considered non toxic, inorganic forms are toxic [26]. In countries such as Bangladesh, China, Hungary, and India, among others, arsenic is found at high concentration in ground water and surface soil [27].

There is strong evidence for a link between bladder cancer and exposure to arsenic (As) in drinking water at concentrations exceeding 300 – 500 µg/l [1,28]; however, there seems to be no elevated risk with As concentrations below 200 µg/l [29], except for the smokers [30]. On the other hand, Meliker and Nriagu [1] reported that health risks from As exposure in the 10 – 100 µg/l range are not obvious. Given low-to-moderate concentrations of As in drinking water, other sources of As exposure such as air, food, occupational hazards, and tobacco may be important [1,28,30]. Exposure to other potential bladder carcinogens in drinking water, such as disinfection byproducts or nitrates, along with mediating factors in the diet, such as selenium and zinc, may also prove to be decisive factors [1]. On the other hand, Kurttio et al. [31] reported in a Finnish study that in spite of very low As exposure levels (0.5 µg/l) they found evidence of a link between As and bladder cancer risk and provided evidence for synergistic effects of smoking and nutritional factors with As.

The mechanism of arsenic-induced bladder cancer remains unclear. Arsenic inhibits indirectly sulfhydryl containing enzymes and interferes with cellular metabolism such as cytotoxicity, genotoxicity and inhibition of enzymes with antioxidant function [32]. On the other hand, the p53 protein may be involved in the development of bladder cancer. In a Taiwanese study, bladder tumours from people chronically exposed to As showed mutations in the p53 gene at codon 175 and transitions at points 9 and 10 [30]. However, in a South American study, Moore et al. [33] did not find evidence that As exposure was associated with an increased prevalence of p53 mutations or immunopositivity of p53 protein in bladder cancer.

There is also evidence that the process of arsenic carcinogenesis may be modulated by genetic differences in DNA repair [34]. Andrew et al (2009) [34] observed gene-environment interaction of 549 controls and 342 cases with arsenic exposure in relation to bladder cancer risk for a variant allele of the double-strand break repair gene XRCC3 T241M (odds ratios (OR) adjusted for age, gender, smoking status (former and current): OR 2.8 (1.1 – 7.3)).

### Occupational exposure

#### Aromatic amines

The most notable risk factor for the development of bladder cancers is occupational exposure to aromatic amines, first noted in England over 100 years ago. The compounds 2-naphthylamine, 4-aminobiphenyl and benzidine can be found in the products from the chemical, dye and rubber industries as well as in hair dyes, paints, fungicides, cigarette smoke, plastics, metal and motor vehicle exhaust and pollutant emissions from industrial installations [35-38].

In 1954, Case et al. [39] reported a 200-fold increased bladder cancer risk for English and Welsh workers exposed to 2-naphthylamine. In the cohort study performed on more than 11 000 workers in the rubber industry, elevated standardised mortality ratios (SMR) for bladder cancer were observed for “storage and shipment” (SMR 253; 95 % CI 93-551) and for “general work” in this industry (SMR 159; 95 % CI 92-279) [40].

4-aminobiphenyl, carcinogenic aromatic amine, present as a carcinogenic component of tobacco smoke is also used in the rubber industry. In a study involving 171 workers in the rubber industry, 19 cases of bladder cancer were observed [40].

Benzidine, used in dye production and the rubber industry, has been identified as the most important carcinogenic aromatic amine regarding human bladder damage. 92 of 331 workers of one of the most important industrial facilities in Leverkusen, Germany, who had been exposed to benzidine production before 1967, eventually contracted bladder cancer [40]. In a Chinese cohort study, where 784 workers were exposed to benzidine, a 35-fold increased of bladder cancer risk was observed [41].

Individuals with occupational exposure to hair dyes such as hairdressers and barbers experience enhanced risk of bladder cancer although it is unclear if other lifestyle aspects are responsible for the increase in bladder cancer risk [36]. In a large population-based case-control study in Los Angeles, personal use of hair dyes was assessed according to the types of hair dyes normally used and compared with people who did not use hair dyes [42]. An elevated bladder cancer risk (odds ratio (OR) 1.9; 95 % CI 1.1 -3.3) was claimed for those who used permanent hair dyes at least once a month, for 1 year or longer. The risk increased to 3.3 (95 % CI 1.3 – 8.4) for those who used permanent hair dyes at least once a month for 15 and more years. Hairdressers who performed their jobs for more than 10 years showed a 5-fold increased risk (95 % CI 1.3 – 19.2) [40].

Firefighters exposed to a long list of carcinogens in combustion products including asbestos, polycyclic aromatic hydrocarbons, benzene, lead and aromatic amines have been shown to be at increased risk of developing a number of cancers including multiple myeloma, non-Hodgkin’s lymphoma, prostate cancer, testicular cancer leukemia, skin cancer, malignant melanoma, brain cancer, and cancer of the rectum, colon, stomach, buccal cavity and pharynx [43].

### 4,4'-methylenebis(2-chloroaniline) (MBOCA)

4,4'-methylenebis(2-chloroaniline) (MBOCA) is a synthetic chemical widely used in industry primarily to produce castable polyurethane parts. Most exposure to MBOCA occurs in the workplace; the workers may inhale small particles of MBOCA in the air or absorb MBOCA dust or vapor through the skin [37]. In a Taiwanese study, a total of 70 MBOCA-exposed workers and 92 non-exposed workers were screened for bladder cancer. The study identified a proven bladder carcinoma in MBOCA-manufacturing factories; a worker with suspected malignant cells; and a worker with atypical cytology combined with gross hematuria. The findings of this study support the conclusions from other studies that MBOCA is potentially carcinogenic to humans and control measures (such as issuing work clothing that must not be worn at home; requiring to shower at the end of the work shift; and improving the ventilation system) are needed to prevent overexposure from inhalation and skin absorption [37].

It has been estimated that the occupational exposures are responsible for 18 % of bladder cancer cases. 2 years' exposure may be sufficient to increase this risk, but the time between exposure and subsequent cancer may be several decades [37].

## Conclusion

In this review, we investigated the associations of the environmental risk factors with bladder cancer. Smoking, in particular cigarette smoking, is a well known risk factor for various diseases including bladder cancer. The effects of other type of smoking (cigar, pipe, Egyptian waterpipe, smokeless tobacco and environmental tobacco smoking) have been investigated only in a few studies. The precise mechanism of smoking-induced bladder cancer has yet to be determined. Another factor strongly associated with bladder cancer is exposure to arsenic in drinking water at concentrations higher than 300 µg/l. Other sources of arsenic exposure such as air, food, occupational hazards, and tobacco may be important, but there are not enough studies to allow demonstration of a link. The mechanism of arsenic-induced bladder cancer remains unclear. The most notable risk factor for the development of bladder cancers is occupational exposure to aromatic amines (2-naphthylamine, 4-aminobiphenyl and benzidine) and 4,4'-methylenebis(2-chloroaniline) that can be found in the chemical, dye and rubber industries as well as in hair dyes, paints, fungicides, cigarette smoke, plastics, metal and motor vehicle exhaust. Although the number of chemicals related to occupational exposure is still growing, it is worth noting that it may take several years or decades between exposure and the subsequent cancer.

## Competing interests

The authors declare that they have no competing interests.

## Authors' contributions

SL and AM conceived and designed the review, collected the data and drafted the manuscript. AS, MD, KV and CM contributed to the conception of the review and its design. AB is a HENVINET project coordinator and contributor to Framework development. All authors read and revised the final manuscript.
